# Nephrogenic Ascites: An Unusual Culprit of Refractory Ascites in a Hemodialysis Patient—A Case Report and Review of Literature

**DOI:** 10.7759/cureus.30876

**Published:** 2022-10-30

**Authors:** Mohammed S Abdalla, Eltaib Saad, Monzer Abdalla, Tasneem I Musa, Khalid Mohamed

**Affiliations:** 1 Internal Medicine, Ascension Saint Francis Hospital, Evanston, USA; 2 Internal Medicine, International University of Africa, Khartoum, SDN

**Keywords:** renal transplantation, hemodialysis, end-stage renal disease, dialysis-associated ascites, nephrogenic ascites

## Abstract

Nephrogenic ascites or dialysis-associated ascites is a rare condition that develops in patients with end-stage renal disease (ESRD) who have been on long-term hemodialysis. It is characterized by rapidly accumulating ascites that is often recurrent and resistant to standard treatment. The diagnosis typically requires the exclusion of common causes of ascites including possible hepatic, cardiac, malignant, and infectious etiologies. Nephrogenic ascites generally has a poor prognosis. Renal transplantation is the sole definitive cure for this difficult-to-treat clinical entity, however, majority of the affected patients are usually deemed unsuitable candidates for transplantation.

In this communication, the authors presented a rare case of nephrogenic ascites that posed a therapeutic challenge in an ESRD patient on regular hemodialysis along with a brief review of the literature regarding the pathogenesis, clinical features, and outcome of nephrogenic ascites.

## Introduction

Nephrogenic ascites is a unique type of ascites that develops in end-stage renal disease (ESRD) patients, especially those undergoing long-term hemodialysis [1, 2]. It represents an infrequent cause of ascites in this population associated with a poor prognosis with a mortality rate of almost 45% within 15 months of diagnosis [1]. Kidney transplantation is the only definitive treatment and often leads to complete resolution of ascites within a few weeks, however, most of these patients are usually deemed unsuitable candidates for renal transplantation [1-3]. In this report, the authors present a case of nephrogenic ascites as a culprit of treatment-refractory ascites in a hemodialysis patient and reviewed the literature on pathogenesis, clinical presentation, and outcome of this uncommon entity that may be encountered at dialysis centers.

## Case presentation

A 63-year-old male with ESRD on regular hemodialysis (three times/week) presented to the emergency department from a skilled nursing facility because of worsening abdominal distension. He has been noticed to be frequently hypotensive over the last few months. His abdominal distension started about four months prior to his presentation. Two months prior to this index presentation, he was treated for symptomatic ascites with the removal of six liters of ascitic fluid. He has been on regular hemodialysis three times weekly, however, fluid removal has been limited due to frequent intradialytic hypotension. His abdomen, however, has become progressively distended during the week leading to his current presentation. Abdominal ultrasound in the nursing facility showed moderate to large ascites. He reportedly missed his last hemodialysis session because of recurrent episodes of hypotension. Past medical history was significant for coronary artery disease (CAD) status post coronary artery bypass graft (CABG) with a challenging post-operative course that was complicated by pulseless electrical activity (PEA) cardiac arrest and failure of liberation from mechanical ventilation due to tracheal stenosis. He became ventilator-dependent and underwent a tracheostomy and percutaneous gastrostomy (PEG) tube placement. The patient’s prior medical history also included ESRD secondary to diabetic and hypertensive nephropathy. He underwent a renal transplant five years prior to index admission but developed transplant failure from septic shock after three years of the transplantation has become anuric and remained on regular hemodialysis three times weekly since then. He also has a medical history of paroxysmal atrial fibrillation, hypothyroidism, and secondary hyperparathyroidism.

At the initial presentation, the patient was cachectic but was not in apparent distress. He was pale but anicteric and has no palpable lymphadenopathy. No peripheral stigmata of chronic liver disease were appreciated. He had a tracheostomy attached to a mechanical ventilator with assisted control mode on minimum ventilatory settings. His vital signs include a blood pressure of 94/51 mmHg, pulse rate of 79 beats per minute, respiratory rate of 21 breaths per minute, and he was afebrile. Cardiac sounds were normal, and no murmurs, gallops, or rubs were heard. His jugular venous pressure was not elevated but has mild pedal edema. He has a functioning right internal jugular tunneled hemodialysis catheter. Abdominal examination revealed a massively distended abdomen, with full flanks and positive fluid thrill. The spleen and liver were not palpable and there were no palpable masses.

**Figure 1:**
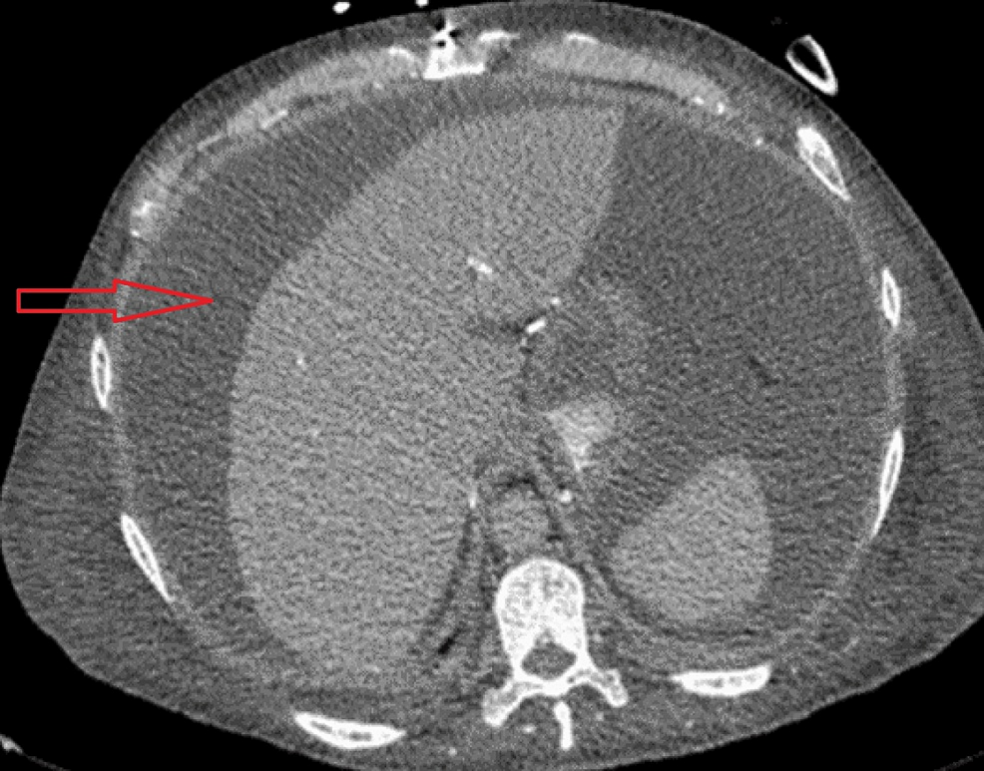
Axial image of contrast-enhanced CT abdomen with massive ascites (horizontal red arrow).

The patient was admitted to the intensive care unit (ICU) for the management of hypotension and ascites. He was treated with vasopressors and was initially treated empirically with broad-spectrum antibiotics for possible septic shock although no clear source of infection has been identified at that time. The hypotension was also thought to be worsened by third-spacing loss due to large ascites. He received scheduled hemodialysis sessions at ICU, but fluid removal was limited by refractory hypotension. Contrast-enhanced computed tomography (CT) of the abdomen and pelvis (Figure 1) revealed a large volume of abdominal and pelvic ascites, the liver was normal in size and contour with no focal lesions and no intrahepatic or extrahepatic biliary dilatation. The spleen was also unremarkable. Kidneys were markedly atrophied and the transplanted kidney was visible in his right hemipelvis with loss of normal contour.

Laboratory workup was remarkable for normocytic anemia (hemoglobin (Hb) of 7.8 g/dl (13.0-17.0 g/dL) and hypoalbuminemia (serum albumin 2.1 g/dl (3.5-5.7 g/dL). Liver function tests (LFTs) were essentially within normal ranges. Virology screening and an extended Acute Hepatitis Panel was entirely negative. His thyroid-stimulating hormone (TSH) was 7.170 (0.27-4.20 uIU/mL) and T4 and Free T4 1.15 ng/dL (0.5-1.6 ng/dL) (on levothyroxine). The rest of the laboratory results are summarized in Table 1.

**Table 1:** Pertinent laboratory results on admission

Laboratory test	Patient’s result	Reference range
White Cells Count (WCC)	11,100/𝜇L	3,000-10,000/𝜇L
Neutrophils percentage	81%	40-65%
C-reactive protein (CRP)	mg/dl	0-7 mg/dl
Serum Creatinine	0.7 mg/dl	0.6-1.3 mg/dl
Blood urea nitrogen (BUN)	17 mg/dl	9.0-25.0 mg/dl
Aspartate aminotransferase (AST)	17 IU/L	13-39 IU/L
Alanine aminotransferase (ALT)	12 IU/L	7-52 IU/L
Alkaline phosphatase	121 IU/L	40-129 IU/L
Total bilirubin	0.5 mg/dl	0.1-1.2 mg/dl
International Randomized Ratio (INR)	1.3	<1.1

The patient’s refractory ascites necessitated several therapeutic percutaneous radiology-guided drainage sessions with the removal of a total of about 14 liters of ascitic fluid. Ascitic fluid analysis showed clear yellow fluid, total ascitic fluid protein of 5 g/dL, an albumin level of 1.7 g/dl, an elevated lactate dehydrogenase (LDH) 101 U/L (<63 U/L), normal triglycerides 32 mg/dl (<65 mg/dL), and a normal amylase 14 U/L. Cell counts were significant of a white cell count (WCC) of 330/cu mm, with only 15% of them were polymorphonuclear neutrophils (PMNs). His calculated serum ascitic albumin gradient (SAAG) was 0.4 g/dL.

Infectious workup from serial cultures ascitic fluid sample was unrevealing. Gram stain and serial bacterial cultures were negative for any growth. Fungal cultures were also negative after four weeks of incubation. Acid-alcohol smear (AFB) smear and mycobacterial culture were both negative after six weeks of incubation. Serial cytology of the ascitic fluid samples was negative for malignant cells.

A transthoracic echocardiogram showed a grossly normal left ventricular cavity size and wall thickness with a left ventricular ejection fraction of 66% (normal range > 50%). The right ventricle was normal in size with normal systolic function, with no evidence of pulmonary valve stenosis or regurgitation. Furthermore, there was no pericardial or pleural effusion. The inferior vena cava was normal in size, with respiratory variation greater than 50%.

At this time a presumptive clinical diagnosis of nephrogenic ascites was made on the basis of a multitude of reasons, including the plausible consideration of the patient’s ESRD status and long-term hemodialysis, the lack of a possible underlying etiology of his ascites after exclusion of possible causes of such a refractory ascites (i.e., hepatic, cardiac, malignant, and infectious culprits), and the supporting evidence of laboratory findings of nephrogenic ascites (a calculated SAAG of 0.4 g/dl (< 1.1 g/dl) and ascitic fluid protein of 5 g/dl).

Hemodialysis was continued during hospitalization, however, hypotension limited fluid removal. He required serial therapeutic paracenteses to relieve symptoms of rapidly accumulating ascites. Unfortunately, the patient was deemed an unsuitable candidate for another renal transplant by the transplant team because of his severe medical co-morbidities and poor functional status.

## Discussion

The pathogenesis of nephrogenic ascites remains poorly understood. At first, it was called dialysis-associated ascites but currently, the term nephrogenic ascites is preferred as ascites development may precede dialysis in many cases [3, 4]. Many factors are felt to be contributing to the pathogenesis of nephrogenic ascites. Malnutrition and hypoalbuminemia were reported to play a major role [3]. Other factors include intra-abdominal fluid shifts secondary to the increased hydrostatic pressure of the hepatic veins and increased permeability of the peritoneal surfaces in part because of uremia [4,5]. Iron and hemosiderin deposition on the peritoneal surfaces have also been postulated as possible contributing pathogenetic factors [6]. Furthermore, chronic kidney disease patients were found to have impaired resorption of peritoneal fluids secondary to obstruction of peritoneal lymphatic drainage [7]. In addition, congestive heart failure, and secondary hyperparathyroidism [8,9] have also been linked to the development of ascites in ESRD patients. Our patient’s parathyroid hormone level was notably elevated, supporting the possibility of hyperparathyroidism as a risk factor.

Pathological examination of the peritoneum in the affected patients usually reveals grossly normal appearing serosal surfaces; however, microscopical examination often shows chronic inflammatory infiltrate and reactive proliferation of the mesothelial cells with variable degrees of fibrosis, nevertheless, the histopathologic examination can be normal in some cases [10,11].

The true incidence of nephrogenic ascites remains unknown. There is marked variability in incidence across different centers (0.7 to 20%), but the incidence may be generally declining owing to improvements in hemodialysis technology with the improving control of volume overload, enhanced dialysis dose, and better nutrition. Interestingly, there is a wide age range of onset (11 to 71 years; mean 42 years) and predilection for the male sex (male:female = 2:1), however, no predilection for a particular race. In 69% of patients, chronic ambulatory peritoneal dialysis preceded hemodialysis [3].

Clinical features of nephrogenic ascites include symptoms of volume overload (i.e., abdominal distension, weight gain, early satiety, and shortness of breath). In addition, patients are typically malnourished and cathectic.

The gross examination of ascitic fluid yields a clear fluid with a relatively higher protein content (3 to 6 g/dL) with a typically low SAAG (<1.1). It also characteristically has a low white blood cell count (<250 cells per mm^3^) [12]. This is in comparison to a SAAG level of >1.1 that characterizes portal hypertension from hepatic causes (for instance liver cirrhosis), or post-hepatic causes (such as hepatic venous occlusion) [13,14]. The diagnosis of nephrogenic ascites thus requires comprehensive evaluation to rule out these important etiologies [10-13].

The initial management of nephrogenic ascites entails the control of volume status with salt and fluid restriction, increased fluid removal via increasing frequency of hemodialysis, intermittent paracentesis for symptomatic ascites, and improved nutritional intake [15]. Hemodialysis, however, may be limited, as in our patient, by the tendency of these patients to develop intradialytic hypotension [3].

Other treatment options involve switching to continuous ambulatory peritoneal dialysis (CAPD) that may reduce the severity of ascites and improve nutritional status if these initial measures fail to provide substantial benefit [3,5,11]. Data suggest that the placement of a peritoneal-venous shunt may result in a reduction of ascites without significant hemodynamic instability [16,17]. However short and long-term complications of the shunt may result in unwanted morbidities [18].

Nephrogenic ascites has a poor prognosis with a mortality rate of 45 percent within 15 months of diagnosis [3]. About one-third of patients develop cachexia [3, 10]. Kidney transplantation is the only definitive treatment with a resulting resolution of ascites within six weeks of transplantation in almost all patients [5,18]. Ascites often recurs after the loss of the transplant function, regardless of the underlying reason for transplant failure, as seen in our reported patient [3,5,16].

## Conclusions

Nephrogenic ascites is an unusual, yet, serious complication of end-stage renal disease that poses a significant therapeutic challenge. The pathogenesis of the entity remains poorly understood. The diagnosis should be suspected in patients with ESRD on long-term dialysis who present with treatment-refractory ascites and typically requires exclusion of other etiologies of exudative ascites. Renal transplantation is the definitive management of this condition, however, many of these patients are not suitable candidates for kidney transplantation.
